# Using the Nintendo™ Wii to Improve Physical Function and Reduce the Risk of Falls in Older Adults: A Randomized Controlled Clinical Trial

**DOI:** 10.3390/s24196358

**Published:** 2024-09-30

**Authors:** María del Carmen Carcelén-Fraile, Agustín Aibar-Almazán, Fidel Hita-Contreras, Marcelina Sánchez-Alcalá, Ana Belén Parra-Díaz, Aday Infante-Guedes, Yolanda Castellote-Caballero

**Affiliations:** 1 Department of Education and Psychology, Faculty of Social Sciences, University of Atlántico Medio, 35017 Las Palmas de Gran Canaria, Spain; 2 Department of Health Sciences, Faculty of Health Sciences, University of Jaén, 23071 Jaén, Spainanabparradi@gmail.com (A.B.P.-D.);; 3 Department of Health Sciences, Faculty of Health Sciences, University of Atlántico Medio, 35017 Las Palmas de Gran Canaria, Spain; aday.infante@pdi.atlanticomedio.es

**Keywords:** balance, muscle strength, flexibility, training, older adults

## Abstract

(1) Background: Numerous exercise programs that improve physical capacity and the risk of falls in older adults have been proposed with varying degrees of success. A novel approach may be to use a video game system that uses real-time force feedback to train older adults. The aim of this study was to evaluate the effects of a Nintendo™ Wii-based exercise program on physical function and risk of falls in older people. (2) Methods: This 12-week randomized controlled clinical trial involved 73 participants: 36 individuals participating in a control group (CG) and 37 in an experimental group (EG) participating in a combined program. Balance was measured using the Tinetti scale, flexibility was assessed with the back scratch test and the sit-and-reach test, and lower body strength was assessed with the 30 s chair stand-up test. (3) Results: The results of this study show significant improvements in balance, gait, flexibility, and strength of the lower limbs compared to a control group. (4) Conclusions: A Nintendo™ Wii-based exercise program for seniors produces improvements in the physical health of older adults. These improvements highlight the importance of integrating physical exercise through video games as an effective strategy to improve the general health and quality of life of older adults.

## 1. Introduction

Aging entails a series of physiological changes that negatively affect physical function and increase the risk of falls in older people [1]. Sarcopenia, defined as the loss of muscle mass and strength, is one of the main causes of functional deterioration in this population [2]. Along with impaired balance and decreased mobility, these problems significantly reduce the quality of life and independence of older adults [3]. With the increasing life expectancy and prevalence of these conditions, it is crucial to develop effective and accessible interventions to mitigate their effects and promote healthy aging [4].

Falls in older people are a major public health problem due to their physical and psychological consequences, which include injuries such as hip fractures, sprains, and bruises [5], as well as a fear of falling and loss of autonomy that can reduce the quality of life of older people by preventing them from carrying out activities of daily living [6]. Each year, 30% of adults aged 65 years and older experience at least one fall, increasing the risk of additional falls in the future [1]. Additionally, falls represent a significant cost to healthcare systems due to hospitalizations and long-term care needs [7]. In this context, the implementation of interventions that can reduce the risk of falls and improve physical function is essential [8]. Impaired balance is one of the greatest risk factors for falls in older people, since, with aging, there is a worsening of balance as a consequence of physical limitations and deterioration of the peripheral sensory system [9].

In recent years, technology has emerged as a promising tool to promote health and well-being among older people [10]. In particular, video game training offers several advantages over traditional exercise methods: (i) it allows users to perform physical activities in a safe and controlled environment, which is especially important for older people at high risk of falls; (ii) interactive games can be more motivating and engaging than conventional exercises, which can lead to greater adherence to the training program; (iii) the accessibility and low cost of video game technology make it a viable option for a wide range of users [11].

Within this type of training, the Nintendo™ Wii, a video game console that uses motion controls, has shown great potential to promote physical activity through interactive games [12]. These games are not only attractive and easy to use, but can also be adapted to individual abilities, offering an innovative and motivating alternative to traditional exercise [13]. The Wii Balance Board, an accessory for the Nintendo™ Wii console, acts similarly to a force platform, allowing users to interact with the game through movement and balance [14]. Several studies have indicated that Wii-based exercise programs can significantly improve mobility, balance, and muscle strength in older people [15,16]. Additionally, the real-time feedback provided by these games can increase users’ motivation and engagement, facilitating longer adherence to the exercise program [17].

Therefore, the present study aims to evaluate the effects of a Nintendo™ Wii-based exercise program on physical function and risk of falls in older people. The hypothesis is that participants who use the Wii Balance Board will experience significant improvements in their balance, mobility, and muscle strength and a reduction in their risk of falls compared to those who do not participate in the program. This study not only contributes to the existing literature on technological interventions for healthy aging but also may offer a practical and economical solution to improve the quality of life of older people.

## 2. Materials and Methods

### 2.1. Study Design

A randomized, controlled clinical trial was carried out to analyze the effects of a Nintendo™ Wii-based exercise program on physical function and risk of falls in older people (NCT06142812). All participants provided informed consent before starting the study, in compliance with the Declaration of Helsinki, good practices, and relevant laws and regulations. The University of Mid-Atlantic Ethics Committee approved the study (CEI/01-010).

### 2.2. Participants

**Figure 1:**
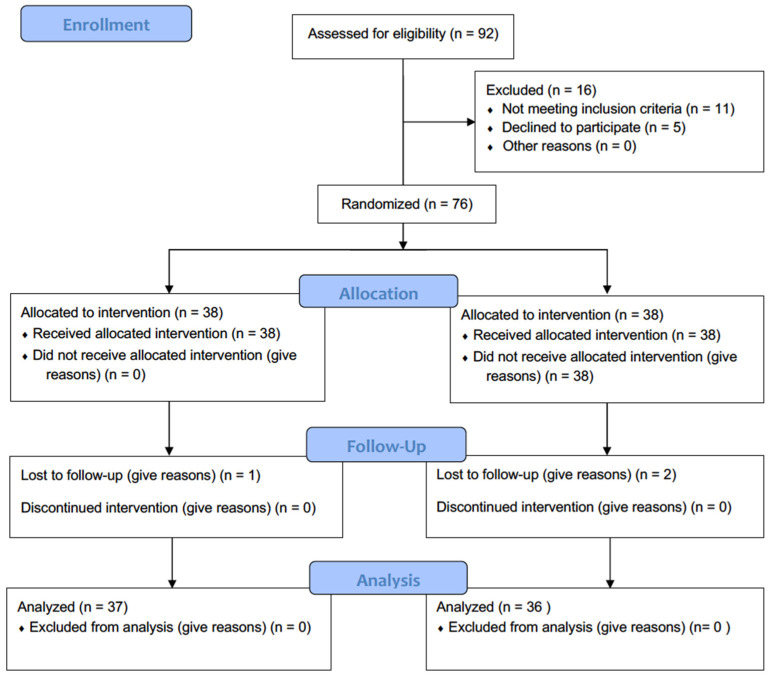
Flowchart of participants in this process.

Of the 92 individuals initially contacted, 76 met the inclusion criteria and agreed to participate in the study (see Figure 1). Participants were recruited through social media ads and posters placed in local community centers frequented by individuals interested in exercise programs. Each person received a detailed explanation of the study and was asked about their willingness to participate. Those who expressed interest were invited to an informational session, where the study requirements, potential benefits, and associated risks were thoroughly discussed. During this session, each participant was also assessed to ensure they met the specific eligibility criteria.

To be eligible to participate, individuals had to (i) be men and women over 65 years of age who voluntarily agreed to participate and were not involved in any additional physical exercise programs and (ii) be able to understand the instructions, respond to the study questionnaires, and participate in the established physical tests. The exclusion criteria included (i) having visual problems that could not be corrected with the use of glasses, contact lenses, or surgery, such as advanced macular degeneration, progressive diabetic retinopathy, advanced glaucoma, and other ocular pathologies that significantly limit central or peripheral vision, and (ii) being enrolled in another physical exercise program for the duration of the study.

### 2.3. Randomization

The allocation of participants was carried out using a series of computer-generated random numbers, equally distributing subjects between the experimental group (EG) and the control group (CG) in a 1:1 ratio. The distribution of the groups was kept secret from the participants, the researchers, and the physical therapist responsible for implementing the intervention. To guarantee this confidentiality, opaque, sealed, and sequentially numbered envelopes were used, which were kept locked away and could only be opened by a person outside the study. At the end of this process, 38 subjects were assigned to the experimental group and 38 to the control group.

### 2.4. Intervention

The Wii Balance Board is a peripheral device for the Nintendo™ Wii console equipped with pressure sensors that detect the user’s center of gravity and balance, allowing interactive exercises in games designed for this purpose.

The intervention group completed a balance training program using the Nintendo™ Wii console and its Wii Balance Board. This program used eight of the nine balance exercises available in the Wii Fit™ game (bubble balance, soccer header, ski jump, table tilt, ski slalom, penguin slide, snowboard slalom, and tightrope). The Zazen exercise was excluded because it involved sitting on the plank, which was difficult for some participants due to problems getting up from and down onto the floor. The participants completed two weekly sessions of 30 min each, over a period of twelve weeks. Groups of four people were formed to perform the exercises simultaneously, under the supervision of instructors trained in a standardized way. During the sessions, participants stood barefoot on the Wii Balance Board, executing the exercises guided by the instructions on the game screen.

The instructor was responsible for operating the consoles, recording data, and organizing the order and duration of the exercises to ensure that all were completed in each session. Although the number of repetitions of each exercise depended on the ability of the participants, the time spent on each exercise was the same for all members of the group. The intervention was considered completed if the participant attended at least 80% of the scheduled sessions.

Members of the control group continued with their daily activities without modifications, receiving guidance to promote physical activity, but were instructed to avoid participating in any type of organized training program and were advised to maintain their usual eating habits. During the 12 weeks of intervention, regular follow-ups were performed through telephone calls to monitor the participants’ physical activity habits.

### 2.5. Outcomes

Data were collected before and after the completion of the intervention phase by a researcher who was fully independent of the intervention and group allocation. Descriptive information, including the participants’ gender, age, educational level, marital status, and employment status, was obtained through self-administered surveys under the supervision of experienced interviewers. Height was measured with an Asimed T201-T4 adult stadiometer (New Delhi, India), while weight was recorded using a Tefal digital scale with an accuracy of 100 g up to 130 kg. These values allowed the body mass index (BMI) to be calculated as the ratio between the weight in kilograms and the height in meters, squared.

#### 2.5.1. Balance

To measure the physical variables of balance, gait, and risk of falls, the Tinetti Scale [18,19] was used. This scale is divided into two parts. One part measures static and dynamic balance with 9 items and a maximum score of 17 points, consisting of (i) balance when sitting: how the person remains seated in a chair without armrests; (ii) getting up from the chair: the patient is asked to get up from the chair without using the arms for support; (iii) attempts to get up: whether more than one attempt is needed to get up; (iv) balance in immediate standing position: how the person maintains balance immediately after getting up; (v) balance in standing position: the person remains standing in a relaxed position for several seconds; (vi) light push on the sternum: a light push is applied to the person’s sternum to evaluate the balance reaction; (vii) balance with eyes closed: the patient is asked to close their eyes while standing; (viii) balance when turning 360 degrees: the patient is asked to turn completely in both directions; and (ix) sitting: how the person sits down again. The other part measures the gait, with a maximum score of 12 points and 7 items taken into account: (i) initiation of walking: how the person begins to walk after receiving the indication to do so; (ii) length and height of each step: the length of the step on each leg (distance between both feet when a step is taken) and the height of the feet (whether the feet are dragged or lifted); (iii) symmetry of each step: whether both steps are similar in length and rhythm; (iv) continuity of each step: whether the person maintains a continuous rhythm when walking; (v) trajectory: the ability to walk in a straight line, without deviating to the sides; (vi) trunk posture: whether the posture is upright or leans forward, to the side, or backward; (vii) foot separation: the separation between the feet when walking, assessing whether it is excessive (greater than 30 cm) or adequate. The sum of the two parts is used to assess the risk of falls. A higher score indicates a lower risk of falls, with 29 being the maximum score achievable. Scores between 19 and 24 points indicate a risk of falls, while scores below 19 indicate high risk.

#### 2.5.2. Flexibility

To assess functional flexibility, the back scratch test (BST) [20] was used for the upper limbs, while the Chair Sit-and-Reach Test (CSRT) [21] was applied for the lower limbs. The BST evaluated shoulder joint flexibility. Participants performed the test while standing, placing one hand behind the head, reaching down the spine, and the other hand behind the lower back, reaching upward. This was done alternately for both sides. The distance between the middle fingertips was recorded, with direct contact considered “zero”. If there was no contact, the gap was noted as a negative value (−), while any overlap was recorded as a positive value (+). The CSRT assessed flexibility in the lower body, specifically targeting the hamstring muscles. Participants sat in a chair positioned against a wall for support and attempted to touch their toes with their hands. A direct touch was marked as “zero”, with any shortfall recorded as a negative value (−) and any reach beyond the toes as a positive value (+).

#### 2.5.3. Strength in the Lower Extremities

This assessment was conducted using the 30 s chair stand test (30s-CST) [22]. Participants started seated in a chair without armrests, maintaining an upright posture with their arms crossed over their chest [23]. They were instructed to stand up and sit down as many times as they could within a 30 s timeframe. A higher number of repetitions indicated better lower body strength.

### 2.6. Sample Size Calculation

The sample size calculation was based on the study by Garcia et al. [24] which investigated the impact of using new technologies, such as interactive gaming consoles, on physical and mental health outcomes in older adults. Assuming a medium effect size of 0.5, a 95% confidence level, and 80% power, a total of 64 participants was required. Anticipating a dropout rate of 15%, the study aimed for 75 participants, divided into 2 groups of 39 each.

### 2.7. Statistical Analysis

The means and standard deviations were calculated for each variable under investigation. Group differences were assessed using Student’s *t*-test for independent samples. To examine the effects of the intervention, a mixed analysis of variance (ANOVA) was performed, with the intervention (EG vs. CG) serving as the intergroup factor and the evaluation time (pre- vs. post-treatment) as the intrasubject factor. The dependent variables included balance, flexibility, and lower limb strength. Specific assessments were conducted for each of these variables. Additionally, the potential interaction between the treatment group and the evaluation period was explored. Cohen’s d was used to estimate the effect size between groups, with values of ≤0.2 indicating a small effect, 0.5 a moderate effect, and 0.8 a large effect [25]. A *p*-value of less than 0.05 was considered statistically significant. Data were analyzed using SPSS software, version 17.0 (SPSS, Inc., Chicago, IL, USA).

## 3. Results

The present study included 28.8% male participants and 71.2% female participants. The subjects participated in at least 97.3% of the scheduled intervention sessions, and no incidents of injury or negative reactions were reported during the intervention period (Table 1).

**Table 1:** Preintervention sociodemographic and clinical characteristics of the participants as a whole and by group.

		Total (n = 73)	Experimental (n = 37)	Control (n = 36)	*p*-Value
Age		70.15 ± 1.76	69.89 ± 1.96	70.42 ± 1.52	0.218
Sex	Male	21 (28.80)	12 (57.10)	9 (42.90)	0.169
	Female	52 (71.20)	25 (48.10)	27 (51.90)	
Occupational Status	Retired	50 (68.50)	27 (54.00)	23 (46.00)	0.107
	Employed	0 (0.00)	0 (0.00)	0 (0.00)	
	Unemployed	23 (31.50)	10 (43.50)	13 (56.50)	
Marital Status	Single	10 (13.70)	6 (16.20)	4 (11.10)	0.816
	Married	41 (56.20)	20 (54.10)	21 (58.30)	
	Divorced/Separated/Widowed	11 (30.60)	11 (29.70)	13 (48.10)	
Educational Status	No education	7 (9.60)	5 (71.40)	2 (28.60)	0.097
	Primary Education	42 (57.50)	23 (54.80)	19 (45.20)	
	Secondary Education	12 (16.40)	5 (41.70)	7 (58.30)	
	University Education	12 (16.40)	4 (10.80)	8 (22.20)	
Height (m)		67.97 ± 11.89	1.66 ± 0.13	1.65 ± 0.14	0.826
Weight (kg)		1.65 ± 0.13	69.23 ± 12.76	66.68 ± 10.95	0.172
BMI (kg/m^2^)		24.87 ± 2.75	25.08 ± 2.70	24.65 ± 2.81	0.778
Waist circumference (cm)		94.49 ± 9.16	96.30 ± 9.99	92.64 ± 7.94	0.203
Hip circumference (cm)		106.64 ± 7.36	107.51 ± 8.07	105.75 ± 6.54	0.618
Waist-to-hip ratio (cm)		0.89 ± 0.06	0.90 ± 0.06	0.88 ± 0.06	0.864
Balance_Tinetti		10.01 ± 2.61	9.89 ± 2.66	10.14 ± 2.59	0.804
Gait_ Tinetti		6.99 ± 2.50	6.78 ± 2.39	7.19 ± 2.61	0.510
Total_Tinetti		17.00 ± 3.87	16.68 ± 3.82	17.33 ± 3.96	0.765
Right Arm_ BST		−10.73 ± 10.83	−12.86 ± 10.97	−8.53 ± 10.38	0.567
Left Arm_ BST		−13.25 ± 11.17	−14.68 ± 9.71	−11.78 ± 12.47	0.101
Right Leg_CSRT		−7.27 ± 9.25	−8.70 ± 10.28	−5.81 ± 7.93	0.054
Left Leg_CSRT		−6.64 ± 9.29	−7.92 ± 9.74	−5.33 ± 8.75	0.474
Lower Body Strength_30s-CST		16.13 ± 2.81	16.35 ± 2.72	15.22 ± 2.53	0.520

Data are expressed as means and standard deviations. Qualitative variables are presented as frequencies and percentages. CG: control group; EG: experimental group; BST: back scratch test; CSRT: Chair Sit-and-Reach Test; 30s-CST: 30 s chair stand test.

### 3.1. Balance

**Figure 2:**
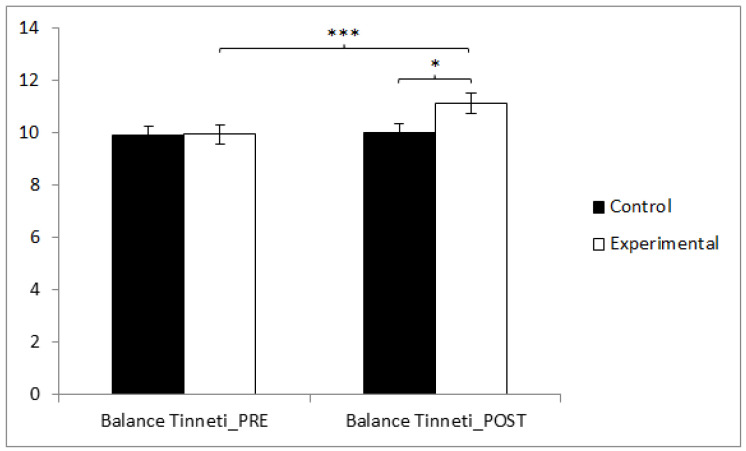
Inter- and intragroup comparisons regarding balance. *** *p* < 0.05, * *p* < 0.001.

According to our findings, in balance, statistically significant differences were found between the pre and post measurements in the training group (t(36) = −5.834, *p* = 0.000, Cohen’s d = 0.42), and statistically significant differences were found between both groups in the post-intervention measure (t(71) = −2.043, *p* = 0.045, Cohen’s d = 0.44) (Figure 2).

**Figure 3:**
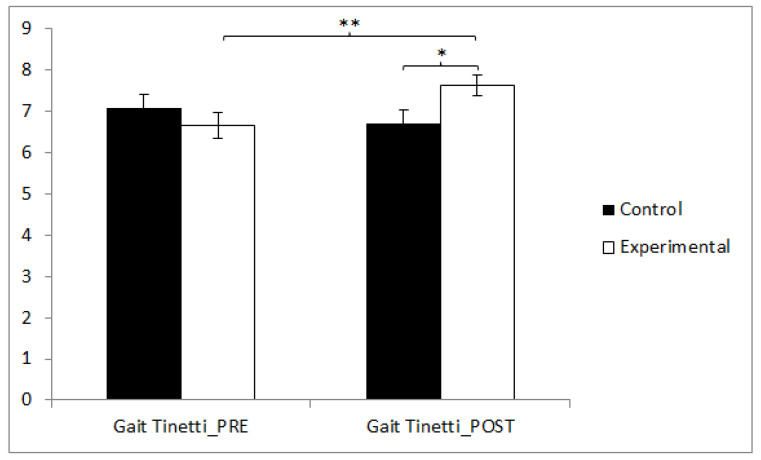
Inter- and intragroup comparisons regarding gait. * *p* < 0.05, ** *p* < 0.01.

Regarding gait, statistically significant differences were found between the pre and post measurements in the treatment/training group (t(36) = −3.220, *p* = 0.003, Cohen’s d = 0.16), and statistically significant differences were found between both groups in the post-intervention measure (t(71) = −2.111, *p* = 0.038, Cohen’s d = 0.10) (Figure 3).

**Figure 4:**
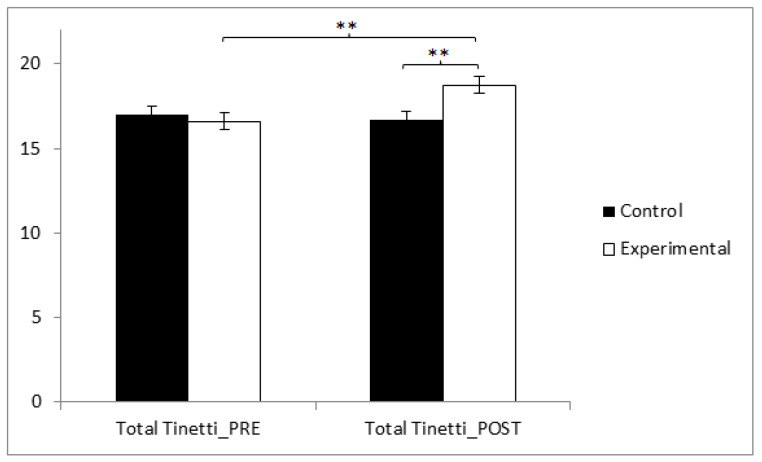
Inter- and intragroup comparisons regarding the total Tinetti score. ** *p* < 0.01.

Finally, concerning the total Tinetti scale score, reflecting fall risk, statistically significant differences were found between pre and post measurements in the treatment/training group (t(36) = −3.088, *p* = 0.004, Cohen’s d = 0.63), and statistically significant differences were found between both groups in the post-intervention measurement: t(71) = −2.769, *p* = 0.007, Cohen’s d = 0.65 (Figure 4 and Table 2).

### 3.2. Flexibility

**Figure 5:**
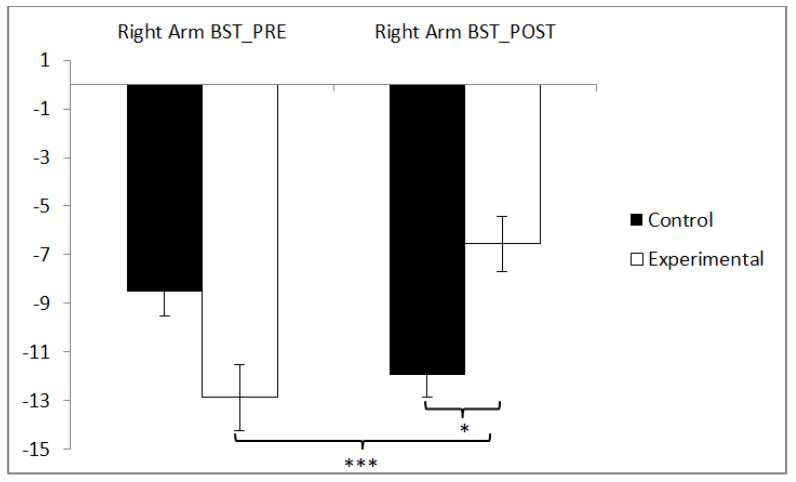
Inter- and intragroup comparisons regarding the right arm. * *p* < 0.05, *** *p* < 0.001.

In the flexibility of the right arm, statistically significant differences were observed between pre and post measurements in the training group (t(36) = −5.141, *p* = 0.000, Cohen’s d = 0.70), and statistically significant differences were found between both groups in the post-intervention measurement (t(71) = −2.047, *p* = 0.044, Cohen’s d = 0.48) (Figure 5).

**Figure 6:**
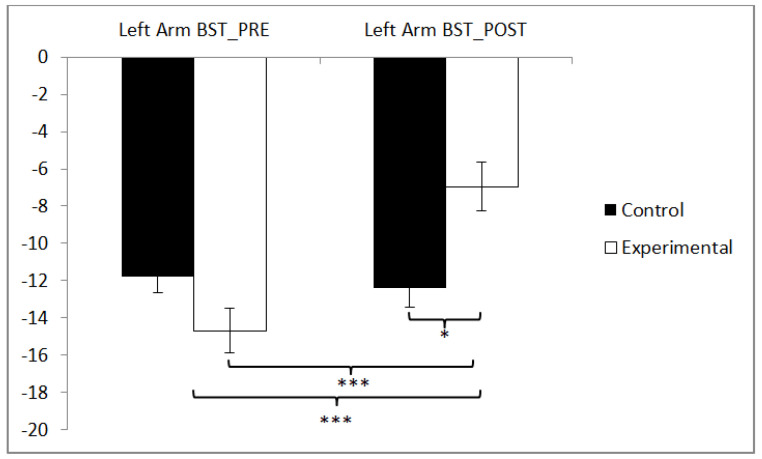
Inter- and intragroup comparisons regarding the left arm. * *p* < 0.05, *** *p* < 0.001.

In the flexibility of the left arm, statistically significant differences were observed between pre and post measurements in the training group (t(36) = −5.938, *p* = 0.000, Cohen’s d = 0.97), and statistically significant differences were found between both groups in the post-intervention measurement (t(71) = −2.423, *p* = 0.018, Cohen’s d = 0.56) (Figure 6).

**Figure 7:**
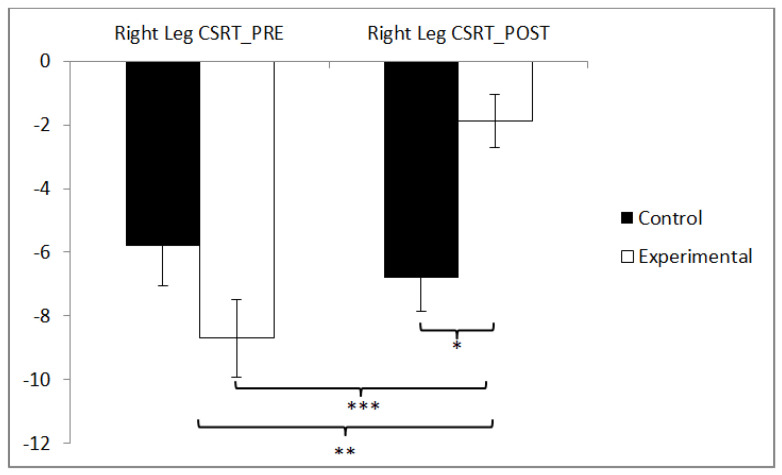
Inter- and intragroup comparisons regarding the right leg. * *p* < 0.05, ** *p* < 0.01, *** *p* < 0.001.

In the flexibility of the right leg, statistically significant differences were observed between pre and post measurements in the training group (t(36) = −5.834, *p* = 0.000, Cohen’s d = 0.78), and statistically significant differences were found between both groups in the post-intervention measurement (t(71) = −2.584, *p* = 0.012, Cohen’s d = 0.60) (Figure 7).

**Figure 8:**
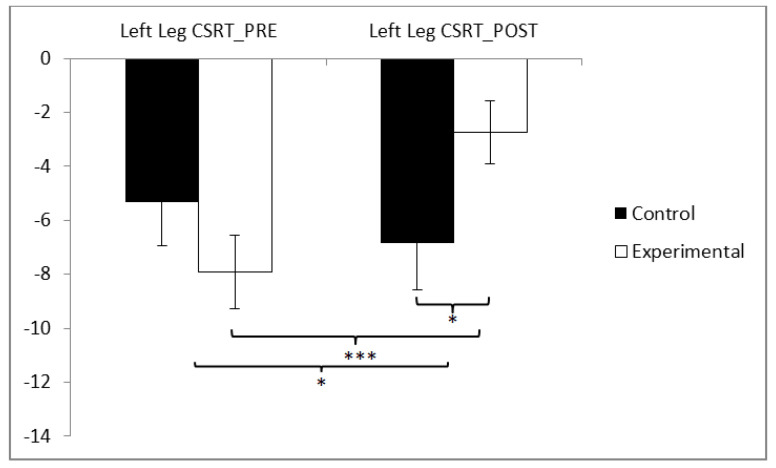
Inter- and intragroup comparisons regarding the left leg. * *p* < 0.05, *** *p* < 0.001.

In the flexibility of the left leg, statistically significant differences were observed between pre and post measurements in the training group (t(36) −4.280, *p* = 0.000, Cohen’s d = 0.63), and statistically significant differences were found between both groups in the post-intervention measurement (t(71) = −2.170, *p* = 0.033, Cohen’s d = 0.50) (Figure 8 and Table 2).

### 3.3. Strength in the Lower Extremities

**Figure 9:**
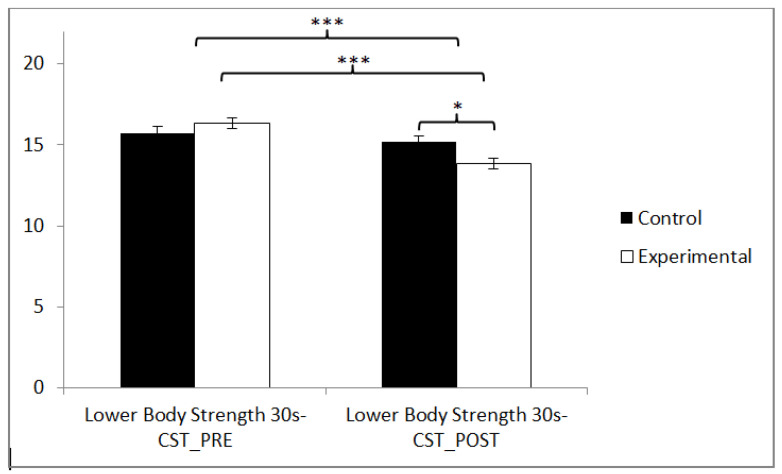
Inter- and intragroup comparisons regarding lower limb strength. * *p* < 0.05, *** *p* < 0.001.

Regarding lower body strength, statistically significant differences were observed between pre and post measurements in the training group (t(36) = 9.117, *p* = 0.000, Cohen’s d = 0.94), and statistically significant differences were found between both groups in the post-intervention measurement (t(71) = 2.295, *p* = 0.025, Cohen’s d = 0.54) (Figure 9 and Table 2).

## 4. Discussion

This study evaluated the effects of a Nintendo™ Wii-based exercise program on improving physical function and risk of falls in older adults. The findings of this study showed that the participants’ balance, flexibility, and limb strength improved after a Nintendo™ Wii-based exercise program when compared to a group that did not undertake any intervention.

Balance is another critical component of physical functionality, especially in older populations [26]. The results of our study show significant improvements in balance after the training intervention. This is particularly relevant since an improvement in balance can contribute to a reduced risk of falls, addressing a common problem in older adults. Wii Fit™ exercises are designed to challenge balance in several ways, which may explain the improvements seen in both our study and that by Bieryla and Dold [16], who found that using interactive Wii Fit™ games significantly improved balance and postural stability in older adults. Their results highlighted the effectiveness of these exercises in training balance in a safe and fun way, although their study had a sample of only 12 older adults. Other studies also found significant improvements but by carrying out another type of intervention, such as the study by Howe et al. [27], who found that a resistance exercise program significantly improved postural stability and reduced the risk of falls in older adults. Furthermore, Ribeiro et al. [28] studied the effects of a balance training program in older adults at high risk of falls and found significant improvements in postural stability and a reduction in the number of falls. Their study used a combination of exercises on stable and unstable surfaces to challenge the balance system, suggesting that variety in exercises may be beneficial. On the other hand, research by Granacher et al. [29] demonstrated that balance training programs that include strength and resistance exercises are effective in improving postural stability and reducing the risk of falls in older populations, underscoring the importance of a multidimensional approach in exercise training to obtain optimal benefits.

Flexibility plays an essential role in the functional capacity of older people in carrying out daily activities and, consequently, in their functional autonomy [30]. Maintaining good flexibility contributes to preventing injuries and improving quality of life in this population [31]. Furthermore, greater flexibility is associated with reduced pain and better overall mobility, facilitating greater participation in physical and social activities [32]. Our findings revealed that a Nintendo™ Wi-based training program produced considerable improvements in lower body strength as assessed by the 30s-CST, with significant intergroup differences post-intervention. Comparing these results with the existing literature, we found that similar studies have found positive effects on the flexibility variable but by carrying out another type of intervention. For example, García-Hermoso et al. [33] demonstrated that a stretching and yoga program in older adults significantly improved flexibility in multiple joints. Similarly, research by Youkhana et al. [34] found that flexibility-based exercise programs not only improve range of motion but also contribute to a better perception of general well-being and reduced musculoskeletal pain. Furthermore, a systematic review by Thomas et al. [35] concluded that flexibility interventions have positive effects on the mobility and functionality of older individuals. This review included several types of training programs, from yoga to static stretching, and found consistent improvements in flexibility and mobility. The results of our study are consistent with these findings, indicating that the improvement in flexibility can be attributed to the structured nature of the training program implemented.

Strength in the lower extremities is essential for maintaining mobility and preventing falls in older populations [36]. Furthermore, strengthening these muscles can significantly improve quality of life and functional independence in older adults [37]. The training program also resulted in significant improvements in lower extremity strength. This result is consistent with studies that have used balance platforms and interactive exercises. A study by Karatrantou et al. [38] evaluated the impact of a Wii Fit™ exercise program on muscle strength and found significant improvements in leg strength and postural stability. The dynamic nature of the exercises, which include jumping and balancing movements, can contribute to the development of muscle strength through repeated, controlled activation of the leg muscles. Furthermore, a study by Donath et al. [39] showed that exercises with unstable platforms, such as the Wii Balance Board, can improve strength and stability by continually challenging the user’s balance. This type of training not only strengthens muscles but also improves coordination and neuromuscular response, essential aspects for maintaining strength and preventing falls in older populations. These results are consistent with studies that have used other types of training, such as the study by Zhao et al. [40] that evaluated the impact of an 8-week resistance training program in older adults and found significant improvements in lower extremity strength and functional capacity. Their study highlighted the importance of progressive resistance training for the prevention of sarcopenia and the improvement of quality of life. Furthermore, a meta-analysis by Beaudart et al. [41] reviewed the effectiveness of different exercise programs in improving muscle strength in older adults. The results indicated that resistance programs, especially those that incorporate high-intensity exercises, are the most effective in increasing muscle strength. This meta-analysis highlights the need to adapt training programs to individual capabilities to maximize benefits.

Among the strengths of our study, we can mention its controlled, randomized, and blind trial design, its large sample size, and its high rate of adherence to the interventions. However, only short-term effects were evaluated. Furthermore, the present study was conducted among older people living in a community, and its conclusions cannot be extended to the entire population. Future studies should be conducted that consider medium- and long-term effects.

## 5. Conclusions

The results of this study support the effectiveness of balance training programs with Nintendo™ Wii in improving flexibility, strength, and balance. These findings are consistent with the current literature, which shows that interactive exercise programs can lead to significant improvements in physical functionality. Future research should focus on optimizing training protocols to maximize benefits and exploring the underlying mechanisms that contribute to these improvements.

## Figures and Tables

**Table 2:** Effects of a psychomotor and cognitive stimulation program on physical capacity.

	EG (n = 37)		CG (n = 36)		Group			Time			Group × Time		
	Pre	Post	Pre	Post	F(71)	*p*-Value	η^2^	F(71)	*p*-Value	η^2^	F(71)	*p*-Value	η^2^
Balance_Tinetti	9.89 ± 2.66	11.08 ± 2.98	10.14 ± 2.59	9.78 ± 2.44	0.98	0.326	0.014	1.592	0.211	0.022	5.580	0.021	0.073
Gait_Tinetti	6.78 ± 2.39	7.19 ± 2.62	8.05 ± 2.20	6.94 ± 2.29	0.499	0.482	0.007	3.966	0.050	0.053	8.805	0.004	0.110
Total_Tinetti	16.68 ± 3.82	19.14 ± 3.95	17.33 ± 3.96	16.72 ± 3.48	1.349	0.249	0.019	3.843	0.054	0.051	10.605	0.002	0.130
Right Arm_BST	−12.86 ± 10.97	−6.54 ± 6.61	−8.53 ± 10.38	−11.94 ± 14.60	0.055	0.816	0.001	1.502	0.224	0.021	16.860	0.000	0.192
Left Arm_BST	−14.68 ± 9.71	−6.95 ± 5.64	−11.78 ± 12.47	−12.39 ± 12.41	0.298	0.587	0.004	25.388	0.000	0.263	34.854	0.000	0.329
Right Leg_CSRT	−8.70 ± 10.28	−1.89 ± 6.82	−5.81 ± 7.93	−6.81 ± 9.28	0.301	0.585	0.004	11.363	0.001	0.138	20.531	0.000	0.224
Left Leg_CSRT	−7.92 ± 9.74	−2.73 ± 6.38	−5.33 ± 8.75	−6.86 ± 9.61	0.169	0.683	0.002	5.399	0.023	0.071	18.171	0.000	0.204
Lower Body Strength_30s-CST	16.35 ± 2.72	13.84 ± 2.62	15.72 ± 2.63	15.22 ± 2.53	0.465	0.497	0.007	31.776	0.000	0.309	14.186	0.000	0.167

Quantitative variables are presented as means and standard deviations. EG: experimental group; CG: control group; BST: back scratch test; CSRT: Chair Sit-and-Reach Test; 30s-CST: 30 s chair stand test; η^2^: eta squared.

## Data Availability

Data are contained within the article.

## References

[1] Montero-Odasso M., Van Der Velde N., Martin F.C., Petrovic M., Tan M.P., Ryg J., Aguilar-Navarro S., Alexander N.B., Becker C., Blain H. (2023). World guidelines for falls prevention and management for older adults: A global initiative. Age Ageing.

[2] Cruz-Jentoft A.J., Bahat G., Bauer J., Boirie Y., Bruyère O., Cederholm T., Cooper C., Landi F., Rolland Y., Sayer A.A. (2010). Sarcopenia: European consensus on definition and diagnosis. Age Ageing.

[3] Xing L., Bao Y., Wang B., Shi M., Wei Y., Huang X., Dai Y., Shi H., Gai X., Luo Q. (2023). Falls caused by balance disorders in the elderly with multiple systems involved: Pathogenic mechanisms and treatment strategies. Front. Neurol..

[4] Fekete M., Szarvas Z., Fazekas-Pongor V., Feher A., Csipo T., Forrai J., Dosa N., Peterfi A., Lehoczki A., Tarantini S. (2022). Nutrition Strategies Promoting Healthy Aging: From Improvement of Cardiovascular and Brain Health to Prevention of Age-Associated Diseases. Nutrients.

[5] Vaishya R., Vaish A. (2020). Falls in Older Adults are Serious. Indian J. Orthop..

[6] Nguyen L.H., Thu Vu G., Ha G.H., Tat Nguyen C., Vu H.M., Nguyen T.Q., Tran T.H., Pham K.T.H., Latkin C.A., Xuan Tran B. (2020). Fear of Falling among Older Patients Admitted to Hospital after Falls in Vietnam: Prevalence, Associated Factors and Correlation with Impaired Health-Related Quality of Life. Int. J. Environ. Res. Public Health.

[7] Florence C.S., Bergen G., Atherly A., Burns E., Stevens J., Drake C. (2018). Medical Costs of Fatal and Nonfatal Falls in Older Adults. J. Am. Geriatr. Soc..

[8] Sherrington C., Fairhall N.J., Wallbank G.K., Tiedemann A., Michaleff Z.A., Howard K., Clemson L., Hopewell S., Lamb S.E. (2019). Exercise for preventing falls in older people living in the community. Cochrane Database Syst. Rev..

[9] Bednarczuk G., Rutkowska I. (2022). Factors of balance determining the risk of falls in physically active women aged over 50 years. PeerJ.

[10] Bertolazzi A., Quaglia V., Bongelli R. (2024). Barriers and facilitators to health technology adoption by older adults with chronic diseases: An integrative systematic review. BMC Public Health.

[11] Bock B.C., Dunsiger S.I., Ciccolo J.T., Serber E.R., Wu W.C., Tilkemeier P., Walaska K.A., Marcus B.H. (2019). Exercise Videogames, Physical Activity, and Health: Wii Heart Fitness: A Randomized Clinical Trial. Am. J. Prev. Med..

[12] George A.M., Rohr L.E., Byrne J. (2016). Impact of Nintendo Wii Games on Physical Literacy in Children: Motor Skills, Physical Fitness, Activity Behaviors, and Knowledge. Sports.

[13] Li Y., Chen D., Deng X. (2024). The impact of digital educational games on student’s motivation for learning: The mediating effect of learning engagement and the moderating effect of the digital environment. PLoS ONE.

[14] Laufer Y., Dar G., Kodesh E. (2014). Does a Wii-based exercise program enhance balance control of independently functioning older adults? A systematic review. Clin. Interv. Aging.

[15] Liu H., Xing Y., Wu Y. (2022). Effect of Wii Fit Exercise with Balance and Lower Limb Muscle Strength in Older Adults: A Meta-Analysis. Front. Med..

[16] Bieryla K.A., Dold N.M. (2013). Feasibility of Wii Fit training to improve clinical measures of balance in older adults. Clin. Interv. Aging.

[17] Xu L., Shi H., Shen M., Ni Y., Zhang X., Pang Y., Yu T., Lian X., Yu T., Yang X. (2022). The Effects of mHealth-Based Gamification Interventions on Participation in Physical Activity: Systematic Review. JMIR Mhealth Uhealth.

[18] Tinetti M.E. (1986). Performance-oriented assessment of mobility problems in elderly patients. J. Am. Geriatr. Soc..

[19] Salvà A., Bolibar I., Lucas R., Rojano-Luque X. (2005). Utilización del POMA en nuestro medio para la valoración del equilibrio y la marcha en una población de personas mayores residentes en la comunidad. Rev. Esp. Geriatr. Gerontol..

[20] Rikli R.E., Jones C.J. (1999). Development and validation of a functional fitness test for community-residing older adults. J. Aging Phys. Act..

[21] Rikli R.E., Jones C.J. (2013). Development and validation of criterion-referenced clinically relevant fitness standards for maintaining physical independence in later years. Gerontologist.

[22] Jones C.J., Rikli R.E., Beam W.C. (1999). A 30-s chair-stand test as a measure of lower body strength in community-residing older adults. Res. Q. Exerc. Sport.

[23] Küçük F., Kara B., Poyraz E.Ç., İdiman E. (2016). Improvements in cognition, quality of life, and physical performance with clinical Pilates in multiple sclerosis: A randomized controlled trial. J. Phys. Ther. Sci..

[24] García M., Rodríguez P., Martínez E. (2021). Effectiveness of New Technologies in Improving Physical and Mental Health of Older Adults: A Randomized Controlled Trial. J. Aging Technol..

[25] Cohen J. (1992). A power primer. Psychol. Bull..

[26] Nascimento M.D.M., Gouveia É.R., Marques A., Gouveia B.R., Marconcin P., França C., Ihle A. (2022). The Role of Physical Function in the Association between Physical Activity and Gait Speed in Older Adults: A Mediation Analysis. Int. J. Environ. Res. Public Health.

[27] Howe T.E., Rochester L., Jackson A., Banks P.M., Blair V.A. (2019). Exercise for improving balance in older people. Cochrane Database Syst. Rev..

[28] Ribeiro F., Gomes D., Laranjo L., Gonçalves L., Jorge F. (2021). The effect of a balance training program on postural control in older adults: A systematic review. Arch. Gerontol. Geriatr..

[29] Granacher U., Gollhofer A., Hortobágyi T., Kressig R.W., Muehlbauer T. (2018). The importance of trunk muscle strength for balance, functional performance, and fall prevention in seniors: A systematic review. Sports Med..

[30] Stathokostas L., McDonald M.W., Little R.M., Paterson D.H. (2013). Flexibility of older adults aged 55-86 years and the influence of physical activity. J. Aging Res..

[31] Nogueira W., Britto R., de Araújo D.S.M., Ferreira R.M. (2019). Flexibility, functional autonomy and quality of life (QoL) of the elderly. Arch. Gerontol. Geriatr..

[32] American College of Sports Medicine (ACSM) (2018). ACSM’s Guidelines for Exercise Testing and Prescription.

[33] García-Hermoso A., Ramírez-Vélez R., García-Alonso Y., Alonso-Martínez A.M., Izquierdo M. (2020). Effects of exercise on functional capacity and quality of life of older adults with frailty: A systematic review and meta-analysis. J. Am. Med. Dir. Assoc..

[34] Youkhana S., Dean C.M., Wolff M., Sherrington C., Tiedemann A. (2016). Yoga-based exercise improves balance and mobility in people aged 60 and over: A systematic review and meta-analysis. Age Ageing.

[35] Thomas E., Battaglia G., Patti A., Brusa J., Leonardi V., Palma A., Bellafiore M. (2019). Physical activity programs for balance and fall prevention in elderly: A systematic review. Medicine.

[36] Dionyssiotis Y. (2012). Analyzing the problem of falls among older people. Int. J. Gen. Med..

[37] Reid K.F., Fielding R.A. (2012). Skeletal muscle power: A critical determinant of physical functioning in older adults. Exerc. Sport. Sci. Rev..

[38] Karatrantou K., Gerodimos V., Dipla K., Zafeiridis A., Kellis E. (2020). Effects of a balance training program on postural control in children. J. Strength Cond. Res..

[39] Donath L., Roth R., Rueegsegger P., Faude O. (2016). Effects of virtual reality training (exergaming) compared to traditional balance training in fall prevention and functional outcomes: A randomized controlled trial. BMC Geriatr..

[40] Zhao R., Zhao M., Xu Z. (2018). The effects of a resistance training program on muscle strength and functional fitness in older adults. Aging Clin. Exp. Res..

[41] Beaudart C., Dawson A., Shaw S.C., Harvey N.C., Kanis J.A., Binkley N., Reginster J.Y., Chapurlat R., Chan D.C., Bruyère O. (2017). Nutrition and physical activity in the prevention and treatment of sarcopenia: Systematic review. Osteoporos. Int..

